# A Method for Explainable Epileptic Seizure Detection Through Wavelet Transforms Obtained by Electroencephalogram-Based Audio Recordings

**DOI:** 10.3390/s26010237

**Published:** 2025-12-30

**Authors:** Paul Tavolato, Hubert Schölnast, Oliver Eigner, Antonella Santone, Mario Cesarelli, Fabio Martinelli, Francesco Mercaldo

**Affiliations:** 1 Faculty of Computer Science, University of Vienna, 1090 Vienna, Austria; 2 Department of Computer Science and Security, St. Pölten University of Applied Sciences, 3100 Sankt Pölten, Austria; hubert.schoelnast@fhstp.ac.at (H.S.); oliver.eigner@fhstp.ac.at (O.E.); 3 Department of Medicine and Health Sciences “V. Tiberio”, University of Molise, 86100 Campobasso, Italy; antonella.santone@unimol.it; 4 Department of Engineering, University of Sannio, 82100 Benevento, Italy; mcesarelli@unisannio.it; 5 Institute of High Performance Computing and Networking, National Research Council of Italy (CNR), 87036 Rende, Italy; fabio.martinelli@icar.cnr.it

**Keywords:** epilepsy, wavelet, convolutional neural network, deep learning, explainability

## Abstract

Accurate classification of brain activity from electroencephalogram signals is essential for diagnosing neurological disorders such as epilepsy. In this paper, we propose an explainable deep learning method for epileptic seizure detection. The proposed approach converts electroencephalogram signals into audio waveforms, which are then transformed into time–frequency representations using two distinct continuous wavelet transforms, i.e., the Morlet and the Mexican Hat. These wavelet-based spectrograms effectively capture both temporal and spectral characteristics of the electroencephalogram signal data and serve as inputs to a set of convolutional neural network models with the aim to detect seizure activity. To improve model transparency, the proposed method integrates three class activation mapping techniques aimed to visualize the salient regions in the wavelet images that influence each prediction. Experimental evaluation on a real-world dataset emphasizes the efficacy of wavelet-based preprocessing in electroencephalogram signal analysis in prompt epileptic seizure detection, showing an accuracy equal to 0.922.

## 1. Introduction and Related Work

Brain activity classification is vital for the early detection and diagnosis of neurological disorders, as it facilitates timely and targeted medical interventions. Among these disorders, epilepsy, characterized by recurrent and unpredictable seizures, requires special attention, since early recognition of abnormal brain patterns can substantially improve patient outcomes. As a matter of fact, the severity of seizures varies widely, ranging from brief lapses in awareness or mild muscle twitches to severe convulsions and complete loss of consciousness [1]. Affecting over 50 million individuals globally, epilepsy is one of the most prevalent neurological conditions [2]. The sudden and often uncontrollable nature of seizures profoundly impacts the daily lives of affected individuals, diminishing their overall quality of life. Beyond its neurological manifestations, epilepsy is frequently accompanied by psychological and social complications, including heightened risks of anxiety and depression, which further challenge effective disease management. Despite being extensively studied [3,4], epilepsy remains a highly complex condition due to its heterogeneous origins, spanning genetic mutations, traumatic brain injuries, infections, and developmental abnormalities. In many cases, the underlying cause remains elusive, thereby complicating both diagnosis and treatment [5].

Electroencephalography (EEG) is widely regarded as the gold standard for epilepsy diagnosis, offering non-invasive access to electrical brain activity. Distinctive EEG patterns such as spikes, sharp waves, and rhythmic discharges, serve as key indicators of epileptic activity. However, manual interpretation of large-scale EEG recordings is labor-intensive, is subject to human error, and demands specialized expertise. Moreover, the transient and unpredictable nature of seizures often necessitates long-term EEG monitoring, further complicating timely and accurate diagnosis [6].

State-of-the-art research on seizure detection from EEG signals has primarily focused on traditional machine learning techniques [7,8,9,10]. These methods typically rely on handcrafted features extracted through approaches such as wavelet transforms, Fourier analysis, or statistical feature descriptors, followed by classification using algorithms like Support Vector Machines or Random Forests [11,12,13,14,15]. While these approaches have demonstrated reasonable performance in specific scenarios, their dependence on manual feature engineering limits their ability to generalize across diverse EEG patterns and patient populations.

In recent years, deep learning has emerged as an interesting alternative, capable of automatically learning discriminative representations from raw data [16,17,18]. Among these methods, convolutional neural networks (CNNs) have demonstrated remarkable success in image processing tasks and are particularly effective at identifying complex patterns and distinguishing between normal and pathological EEG signals [19]. Authors in Ref. [16] present MamlFormer, a multimodal transformer-based framework that effectively extracts rich features from histopathological images, showing how deep learning models can capture subtle diagnostic patterns without relying on handcrafted descriptors. Researchers in Ref. [17] introduce a robust and explainable deep learning method for cervical cancer screening, further illustrating that convolutional neural networks can successfully learn relevant visual characteristics directly from raw medical images while integrating interpretability techniques such as Grad-CAM, which resonates with the approach adopted in this study, while authors in Ref. [18] propose a causality graph attention network for esophageal pathology grading, confirming the effectiveness of advanced deep models in identifying complex nonlinear patterns and enhancing diagnostic accuracy. Together, these works provide solid evidence that deep learning methods are well-suited for extracting high-level representations in medical analysis tasks, thereby justifying their application in EEG-based seizure detection as explored in this paper.

While CNNs can process both numerical and categorical data, their superior performance in visual pattern recognition makes them especially suitable for EEG analysis [20]. Nonetheless, despite these advances, most existing deep learning models still face challenges related to explainability, and this aspect is crucial for considerations of clinical applicability.

Starting from these considerations, we propose an approach for epileptic seizure detection, where EEG signals, originally numerical in nature, are first converted into audio waveforms and subsequently transformed into time–frequency images using Morlet and Mexican Hat wavelet transforms, which are then used as inputs for CNN models.

Electroencephalogram (EEG) signals are inherently non-stationary and characterized by complex temporal and spectral dynamics that evolve across multiple time scales. Traditional EEG analysis methods often operate directly on raw time-series data or on frequency-domain representations derived through Fourier-based techniques. While effective in many scenarios, such approaches may be limited in their ability to capture transient patterns, subtle rhythmic structures, and cross-frequency interactions that are critical for early seizure detection.

The conversion of EEG signals into audio waveforms is motivated by a well-established body of research on EEG sonification and auditory data display [21,22]. Sonification has been widely explored as a means to preserve and highlight the rich temporal–spectral structure of brain activity by mapping neural signals into the auditory domain [21,23]. This transformation allows EEG signals to be treated as audio signals, thereby enabling the application of mature and highly optimized audio signal processing techniques that are particularly well suited for analyzing non-stationary data.

Auditory-domain analysis offers several advantages. First, audio representations facilitate detailed time–frequency analysis through techniques such as spectrograms and wavelet transforms, which have been shown to effectively capture transient oscillations and rhythmic patterns in EEG data [24]. Second, both perceptual and computational studies have demonstrated that auditory representations are highly sensitive to variations in pitch, timbre, amplitude modulation, and temporal structure, making them suitable for revealing subtle neural dynamics that may be difficult to detect using conventional visual inspection of EEG signals [25]. These properties are particularly relevant for pre-ictal EEG analysis, where early seizure-related patterns may manifest as weak or short-lived spectral changes.

Importantly, transforming EEG signals into audio and subsequently into time–frequency images enables the direct application of convolutional neural networks (CNNs) originally developed for 2-D image recognition. Recent studies have demonstrated that CNNs trained on time–frequency EEG representations significantly outperform traditional machine learning approaches based on handcrafted features in seizure detection and EEG classification tasks [26]. By leveraging this paradigm, the proposed approach benefits from the strong pattern recognition capabilities of deep CNN architectures while maintaining a meaningful physiological interpretation through time–frequency analysis.

Therefore, the EEG-to-audio conversion in our framework should be understood as an intermediate representation that bridges raw EEG signals and image-based deep learning models. This design choice facilitates enhanced visualization of EEG time–frequency dynamics, improved detection of subtle rhythmic and transient patterns associated with pre-epileptic activity, and effective exploitation of high-performance CNN architectures combined with explainability techniques. The proposed pipeline is thus firmly grounded in prior work on EEG sonification, auditory data analysis, and deep learning-based EEG classification.

### 1.1. Contribution of the Study

To address the limitation of deep learning about prediction explainability, the proposed method proposes an explainable approach for seizure detection based on EEG analysis: specifically, it integrates an explainability mechanism designed to highlight the EEG-derived image regions that most strongly influence the predictions of the model.

### 1.2. Research Question and Research Variables

Thus, the aim of the proposed method is to understand if it is possible to consider a binary classification task for distinguishing between two categories: pre-epileptic seizure (i.e., the period preceding seizure onset) and other brain activity.

Moreover the dependent variables are the accuracy, precision, recall, F-Measure, AUC, and execution time, while the independent variables are CNN architecture type and wavelet transform type (i.e., Morlet and Mexican Hat).

The remainder of this paper is structured as follows: Section 2 details the proposed methodology; Section 3 describes the experimental setup and presents the results; and last section concludes the paper with key findings and directions for future research.

## 2. The Method

**Figure 1:**
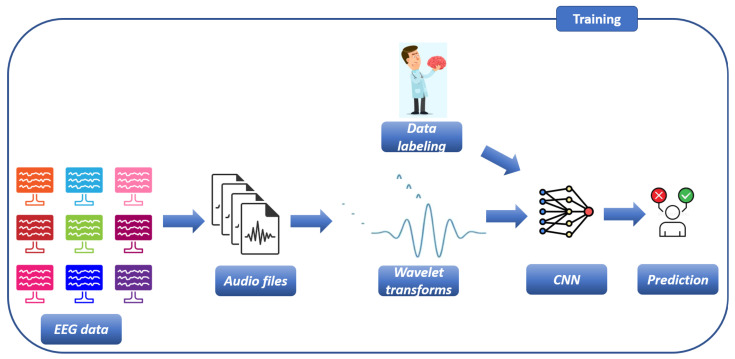
The training step of the proposed method for epilepctic seizure detection and localisation.

**Figure 2:**
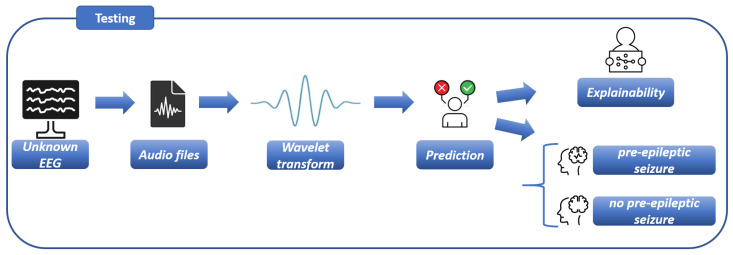
The testing step of the proposed method for epilepctic seizure detection and localisation.

In this section, we present the proposed explainable method for epileptic seizure detection using Morlet and Mexican Hat wavelet transforms obtained starting from EEG-based audio recordings. The overall workflow of the proposed method is shown in Figure 1 and Figure 2, respectively related to the training and the testing step of the proposed method. The motivation behind the conversion of EEG signal into sound wave is to exploit the rich temporal and frequency characteristics of EEG data through auditory domain analysis. Converting EEG signals into sound waves allows us to exploit audio signal processing techniques, such as spectrogram-based feature extraction and auditory perception, inspired transformations which can enhance the representation of non-stationary EEG dynamics. Specifically, this approach facilitates (i) improved visualization of time–frequency patterns [27], (ii) potential identification of subtle rhythmic structures analogous to auditory cues [28], and (iii) the application of deep learning architectures originally optimized for image recognition [29,30].

As a matter of fact, the conversion of EEG signals into sound has been widely explored to preserve and highlight the rich temporal and spectral structure of brain activity through auditory-domain analysis [31,32]. EEG sonification facilitates the use of audio-signal–processing techniques such as spectrograms, time–frequency decompositions, and auditory-inspired feature extraction pipelines that are effective for non-stationary biomedical signals [33,34]. Auditory representations can reveal subtle rhythmic structures and temporal patterns that may remain hard to perceive visually [35,36], leveraging perceptual sensitivity to pitch, timbre, and temporal modulation [37,38]. Transforming EEG into audio and then into spectrogram images enables the direct application of high-performance deep learning architectures originally developed for 2-D image analysis, a strategy already shown effective in EEG classification tasks [39,40]. As highlighted, using time–frequency EEG images as CNN inputs is well-supported by recent literature demonstrating substantial performance improvements in seizure detection and EEG event classification [41,42].

As shown in Figure 1, the process begins with the acquisition of EEG data from patients. Once collected, the EEG signals are converted into corresponding audio files.

For audio file generation, several audio parameters are defined, including the sampling rate (rate = 44,100 Hz), the total signal duration (T = 10 s), and the tone frequency (f = 440 Hz), corresponding to the musical note A4. A time array t is then created using the numpy.linspace function to represent uniformly spaced time samples over the duration of the signal.

The generated waveform is written to disk as a 24-bit WAV file using the wavio.write() function, which takes the waveform array x, the sampling rate, and the bit depth as parameters.

After generating audio recordings from the EEG signals, these audio files are transformed into time–frequency images through the application of Morlet and Mexican Hat wavelet transforms, as depicted in Figure 1.

The wavelet transform is a signal processing technique that enables multi-resolution analysis by decomposing a signal into localized wavelets in both time and frequency. This makes it particularly suitable for analyzing non-stationary and transient signals such as EEG and audio data.

In this paper, continuous wavelet transforms (CWT) are applied using two mother wavelets i.e., Morlet and Mexican Hat, with the aim a to generate complementary time–frequency representations. The Morlet wavelet, characterized by strong time–frequency localization, effectively captures oscillatory signal components. Conversely, the Mexican Hat wavelet, which is the second derivative of a Gaussian function, excels at identifying singularities and sharp transitions. Together, these transforms enhance feature diversity for downstream classification. We exploited the PyWavelets library https://pywavelets.readthedocs.io/en/latest/ (accessed on 23 December 2025) for the wavelet implementation, widespread exploited for research purposes [43,44,45,46].

The Morlet-based CWT function performs a CWT on a one-dimensional signal (in the analysed case an EEG-derived audio) using the Morlet wavelet as the mother function. It computes the wavelet coefficients across a range of scales and time points, producing a matrix that captures how the frequency content evolves over time. The resulting scalogram is saved as an image, representing the Morlet-based time–frequency distribution.

The Mexican Hat-based CWT function applies a CWT using the Mexican Hat wavelet. The resulting wavelet coefficients are visualized as a heatmap, capturing time–frequency variations within the signal. The Mexican Hat wavelet’s sensitivity to discontinuities and sharp transitions complements the Morlet wavelet’s oscillatory feature detection, providing a more complete signal characterization.

We considered EEG segments composed by 100 different measurement. From each EEG-derived audio recording, two plots are generated: the first one using the Morlet wavelet and the second one using the Mexican Hat wavelet. These image sets are organized into two distinct datasets, each serving as input to a collection of convolutional neural network (CNN) models for binary epileptic seizure detection, as depicted in Figure 1.

The proposed method considers ten different CNN architectures i.e.,

- AlexNet: One of the first deep CNNs, consisting of five convolutional and three fully connected layers, which popularized ReLU activation and dropout regularization.
- LeNet: A pioneering CNN architecture designed for digit recognition, composed of two convolutional and two fully connected layers, serving as the foundation for later deep learning models.
- STANDARD_CNN: A custom network comprising three convolutional layers, each followed by max pooling, and two fully connected layers with dropout for regularization.
- MobileNet: A lightweight architecture optimized for efficiency on mobile and embedded devices, utilizing depthwise separable convolutions to minimize computational cost.
- DenseNet: Features dense connectivity, where each layer receives inputs from all preceding layers, promoting feature reuse and improved gradient flow.
- EfficientNetV2: A refined version of EfficientNet that employs compound scaling and progressive learning to achieve high accuracy with reduced training time and memory usage.
- ResNet50: Incorporates residual connections to enable the training of very deep networks by addressing the vanishing gradient problem.
- VGG16: A deep architecture composed of 16 layers using small 3×3 convolutional filters, offering a uniform and simple design that achieves strong performance.
- VGG19: An extended variant of VGG16 with 19 layers, providing enhanced feature extraction capability through increased depth.
- GoogleNet (Inception v1): Utilizes parallel convolutional operations within Inception modules to capture multi-scale features efficiently with reduced parameter count.

The classification task is formulated as a binary problem, assigning each EEG-derived image to one of the following categories:

- PRE: EEG recordings related to a subject with pre-ictal seizures;
- OTHER: EEG recordings related to a brain activity different from pre-ictal seizures.

Figure 2 is related to the testing step of the proposed method, where the model built in the training step (shown in Figure 1) is evaluated.

The final stage of the proposed framework focuses on explainability. After training the CNN models, we interpret their decision-making processes using Class Activation Mapping (CAM) techniques, specifically Gradient-weighted Class Activation Mapping++ [47] (Grad-CAM++), Score-CAM and Score-CAM-Fast. These methods generate heatmaps that highlight the most influential regions within the EEG-derived wavelet images contributing to each classification decision. Employing several CAMs provides complementary perspectives, enhancing the interpretability and trustworthiness of the classification outcomes. As a matter of fact, each of these methods estimates the contribution of distinct spatial regions to the model’s final prediction, thereby offering visual insight into its internal reasoning process. This step is essential for enhancing the interpretability of the model, a crucial requirement for the reliable deployment of deep learning systems in clinical practice.

In the follow we describe the working mechanism for each CAM we considered.

### 2.1. Grad-CAM++

Grad-CAM++ extends the original Grad-CAM by using pixel-wise (spatial) weighting of the gradients, allowing multiple object instances and fine-grained contributions to be better captured. The per-pixel coefficient for feature map *k* at location (i,j) is defined as

(1) $$a_{ij}^{k,c}\;=\;\frac{\frac{\partial^{2}y^{c}}{{\left(\partial A_{ij}^{k}\right)}^{2}}}{\;2\frac{\partial^{2}y^{c}}{{\left(\partial A_{ij}^{k}\right)}^{2}}\;+\;\sum_{a}\sum_{b}A_{ab}^{k}\;\frac{\partial^{3}y^{c}}{{\left(\partial A_{ab}^{k}\right)}^{3}}\;\;},$$

where the sums run over all spatial positions (a,b) of the same feature map $A^{k}$. The final Grad-CAM++ weight for map *k* is obtained by summing the per-pixel coefficients multiplied by the positive part of the first-order gradient:

(2) $$\alpha_{k}^{c}\;=\;\sum_{i}\sum_{j}a_{ij}^{k,c}\;\mathrm{ReLU}\;\left(\frac{\partial y^{c}}{\partial A_{ij}^{k}}\right).$$

The Grad-CAM++ heatmap is then

(3) $$L_{\mathrm{Grad-CAM}++}^{c}\;=\;\mathrm{ReLU}\;\left(\sum_{k}\alpha_{k}^{c}A^{k}\right).$$

where: $y^{c}$: The score (logit) for class *c* produced by the network before applying the softmax function. It represents how strongly the model associates the input image with class *c*.

### 2.2. Score-CAM

Score-CAM is a gradient-free approach that evaluates the influence of each upsampled feature map on the class score through forward passes. Let ${\hat{A}}^{k}\in {\left[0,1\right]}^{H\times W}$ be the *k*-th feature map upsampled to the input resolution. Denote by f(·) the network function returning the pre-softmax score for class *c* given an image *I*. The importance score of each map is

(4) $$s_{k}^{c}\;=\;f_{c}\left(I⊙{\hat{A}}^{k}\right),$$

where: ⊙: The Hadamard (element-wise) product operator, denoting pixel-wise multiplication between two matrices or tensors of the same dimensions. For example, $X⊙\mathrm{Up}(A^{l})$ represents the input image *X* multiplied element-wise with the upsampled activation map $A^{l}$.

Which is normalized to produce weights:

(5) $$w_{k}^{c}\;=\;\frac{\max(0,\;s_{k}^{c})}{\sum_{\ell }\max\left(0,\;s_{\ell }^{c}\right)}.$$

where: *H*, *W*: The height and width of the convolutional feature map $A^{k}$, respectively. Thus, $A^{k}\in R^{H\times W}$, and the indices (i,j) iterate over these spatial dimensions. $s_{c}^{l}$ (or *scl*): The score-based weight assigned to feature map $A^{l}$ for class *c* in Score-CAM. It quantifies the contribution of the feature map to the prediction of class *c* by evaluating the model’s response when the input image is masked by the activation map $A^{l}$.

The Score-CAM heatmap is the weighted combination of the upsampled maps:

(6) $$L_{\mathrm{Score-CAM}}^{c}\;=\;\sum_{k}w_{k}^{c}\;{\hat{A}}^{k}.$$

### 2.3. Score-CAM Fast

Score-CAM Fast reduces computational cost by selecting a subset of feature maps, using approximations, or aggregating similar maps. For a chosen subset K, the weights and heatmap are

(7) $$w_{k}^{c}\;=\;\frac{\max(0,\;s_{k}^{c})}{\sum_{\ell \in K}\max(0,\;s_{\ell }^{c})},\;L_{\mathrm{Score-CAM}(\mathrm{Fast})}^{c}\;=\;\sum_{k\in K}w_{k}^{c}\;{\hat{A}}^{k}.$$

Grad-CAM++, Score-CAM, and Score-CAM Fast all aim to provide class-discriminative visual explanations for convolutional neural networks, but they differ in computational design and localization fidelity. Grad-CAM++ produces sharper heatmaps capturing multiple object instances using higher-order derivatives. Score-CAM evaluates feature map contributions via forward passes and is often less noisy, but computationally expensive. Score-CAM Fast approximates or selects feature maps to reduce cost while maintaining explanation quality.

Table 1 provide a comparison between the three CAMs we considered in the proposed method.

As previously discussed, each CAM technique employs a specific algorithm to highlight the regions that are most influential for a model’s prediction, thereby providing an explanation of the decision-making process. Consequently, when a given wavelet image is provided as input to the model, the different CAM techniques are expected to emphasize similar regions of diagnostic relevance.

## 3. Experimental Analysis

In this section, we present the results of the experimental analysis conducted to evaluate the effectiveness of the proposed method for pre-epileptic seizure detection.

For experimentation, we utilized a publicly available dataset (https://doi.org/10.5281/zenodo.10808054, (accessed on 23 December 2025)), which contains intracranial EEG recordings from 20 patients diagnosed with drug-resistant focal epilepsy. All patients underwent extensive pre-surgical monitoring at the University Hospital of Freiburg, Germany. The dataset provides approximately 24 h of continuous EEG recordings per patient, sampled at 256 Hz from up to 128 intracranial electrodes, offering high temporal resolution and comprehensive spatial coverage of epileptogenic regions.

The EEG data were categorized into four distinct classes: pre-ictal (period preceding seizure onset), ictal (active seizure phase), post-ictal (period immediately following seizure termination), and inter-ictal (seizure-free intervals) [48,49]. Since the primary goal of this study is the early detection of epileptic seizures, the proposed experimental analysis focuses on identifying pre-ictal activity. Accordingly, the classification problem is formulated as a binary task, where the PRE label represents pre-ictal segments, and all remaining categories (ictal, post-ictal, and inter-ictal) are combined into a single non-pre-ictal class.

Each audio file is generated from the aggregated EEG signal across the 128 electrodes, meaning that every wavelet image corresponds to a single measurement derived from all electrode channels. After generating the audio recordings, we produced wavelet-based time–frequency images, yielding two distinct datasets:

- D1: images generated using the Morlet wavelet transform,
- D2: images generated using the Mexican Hat wavelet transform.

**Figure 3:**
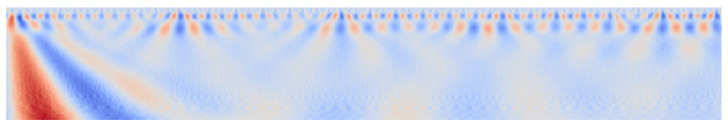
An examples of images related to the D1 dataset obtained through the Morlet wavelet transform, labeled with the PRE label.

**Figure 4:**
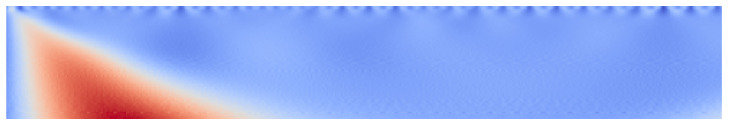
Two examples of images related to the D2 dataset obtained through the Mexican Hat wavelet transform, labeled with the PRE label.

Figure 3 and Figure 4 shows two examples of pre-epileptic (PRE) EEG signals from the D1 and D2 dataset transformed using the Morlet and Mexican Hat wavelet functions, respectively. The color scale represents the signal energy distribution across time and frequency: red regions correspond to high energy or strong activation, whereas blue regions indicate low energy or weak activation. This color encoding highlights time–frequency zones associated with variations in neural activity related to pre-epileptic patterns.

The Morlet wavelet transform (shown in Figure 3) captures the fine-grained oscillatory behavior of the signal, offering enhanced time–frequency localization that highlights transient rhythmic activity often associated with pre-ictal states. The presence of distinct, recurring high-frequency components suggests early neural synchronization patterns that precede seizure onset.

Conversely, the Mexican Hat wavelet transform (shown in Figure 4) produces a smoother and more distributed representation, emphasizing low-frequency variations and energy transitions within the signal. This broader view can be particularly useful for detecting gradual shifts in neural dynamics leading to seizure initiation. The visual contrast between these two wavelet representations underscores how different mother wavelets can reveal complementary aspects of the same pre-epileptic signal, while the Morlet emphasizes temporal precision and oscillatory detail, the Mexican Hat highlights amplitude modulation and overall signal energy distribution.

For both datasets, a total of 4000 wavelet images were generated (2000 per class). Each dataset was divided into training, validation, and testing subsets using an 80:10:10 split ratio, corresponding to 1600 images per class for training, 200 for validation, and 200 for testing.

The Python code we developed for model training, testing and for prediction explainability is freely available for research purposes at the following GitHub repository: https://github.com/FrancescoMercaldo/tami (accessed on 23 December 2025). For code developing we considered the Python programming language 3.9.0. version. The experiment were performed on a machine equipped with Intel Core i9 at 2.25 GHz, 32 GigaBytes of RAM and a NVIDIA GeForce RTX 4070 Laptop GPU with 8 GygaByte as memory as graphic card running on a Microsoft Windows 11 operating system.

Table 2 presents the hyperparameter configurations adopted for the experimental analysis. We consider twenty experiments with the aim to assess the performance of ten CNNs i.e., AlexNet, LeNet, STANDARD_CNN, MobileNet, DenseNet, EfficientNetV2, ResNet50, VGG16, VGG19, and GoogleNet, across two distinct datasets: D1 (composed of Morlet wavelet images) and D2 (composed of Mexican Hat wavelet images).

As depicted in Table 2, we train the models using images of size 224×224×3. The learning rate was fixed at $1\times 10^{-5}$, a value selected after preliminary trials to ensure stable convergence and prevent gradient explosion or premature overfitting. Each model was trained for 10 epochs with a batch size of 16.

These hyperparameter settings were maintained across all experiments to ensure a fair and unbiased comparison among the different architectures. The goal of this uniform configuration was to isolate the effect of network design on classification performance, rather than introducing variability from differing training conditions. The use of both wavelet-based datasets (D1 and D2) further allowed an evaluation of how distinct time–frequency representations influence the models’ ability to detect pre-epileptic activity. We do not considered cross-validation in the experiments.

Table 3 summarizes the results obtained from the experimental analysis, by reporting loss, accuracy, precision, recall, F-measure, area under the ROC curve (AUC), and testing execution time. Each experiment corresponds to a distinct CNN trained on either the D1 (Morlet wavelet) or D2 (Mexican Hat wavelet) dataset, following the uniform hyperparameter configuration described in Table 2.

As shown from Table 3, among the evaluated models, DenseNet and EfficientNetV2 achieved the highest overall performance across both datasets, with DenseNet on D1 reaching an accuracy of 0.922 and an AUC of 0.976, indicating interesting discriminative capability in distinguishing pre-ictal from non-pre-ictal states. MobileNet and ResNet50 also demonstrated competitive performance, achieving accuracies above 0.84 with relatively shorter training times compared to deeper architectures. In contrast, models such as LeNet, VGG16, VGG19, and GoogleNet failed to converge effectively under the given conditions, maintaining baseline-level performance (accuracy approximately equal to 0.5), suggesting limited suitability for this specific classification task without architectural adaptation or parameter tuning.

Execution times varied proportionally with model complexity, ranging from approximately 9 min for lightweight networks (LeNet, STANDARD_CNN) to over 22 min for deeper models such as DenseNet.

Overall, the results confirm that feature-rich architectures (particularly DenseNet and EfficientNetV2) are more capable of capturing the discriminative spatio-temporal features present in wavelet-transformed EEG images, thereby enhancing pre-epileptic seizure detection performance.

**Figure 5:**
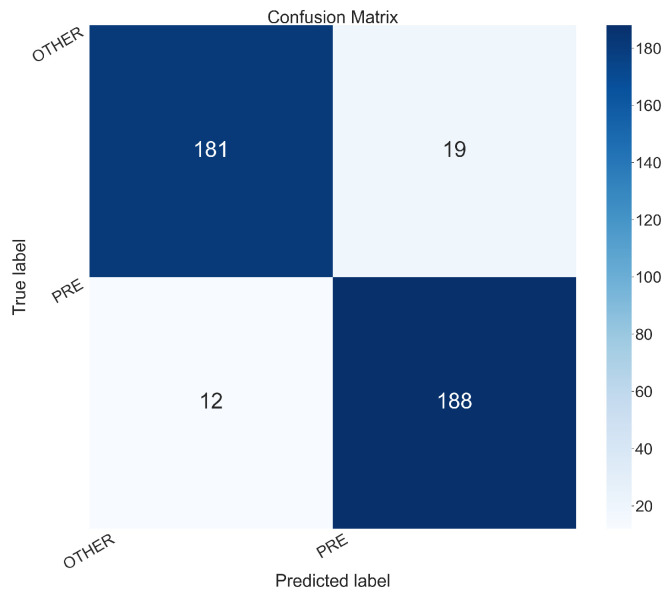
Confusion matrix obtained from the Experiment 5 (the one with the model DenseNet and the D1 dataset).

**Figure 6:**
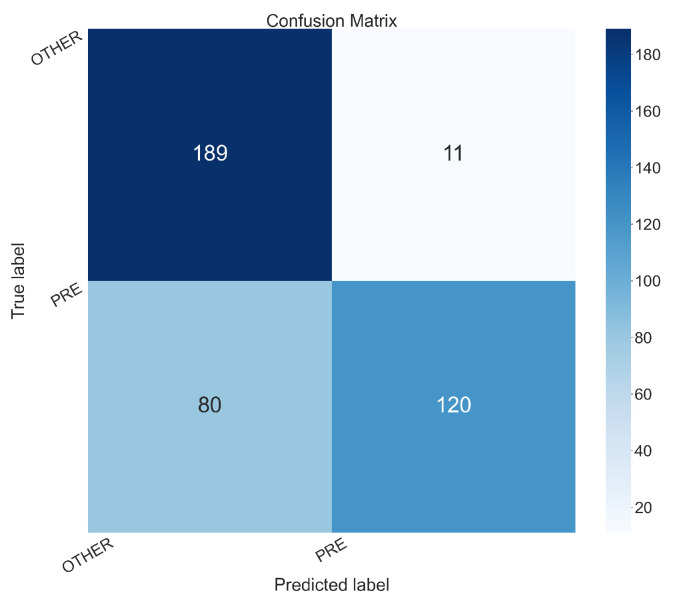
Confusion matrix obtained from the Experiment 16 (the one with the model EfficientNetV2 and the D2 dataset).

To better understand the performances for each class (i.e., PRE and OTHER) in the follow we present the confusion matrices related to the models obtaining the best accuracy for the D1 and D2 dataset i.e., the ones obtained with Experiments 5 (D1 dataset), shown in Figure 5 and 16 (D2 dataset), shown in Figure 6.

The confusion matrix in Figure 5 is related to Experiment 5, which used the DenseNet model on the D1 dataset. It exhibits following values:

- True Positives (PRE correctly predicted as PRE): 188
- True Negatives (OTHER correctly predicted as OTHER): 181
- False Positives (OTHER incorrectly predicted as PRE): 19
- False Negatives (PRE incorrectly predicted as OTHER): 12

The confusion matrix in Figure 5 shows a well-balanced performance. The model is effective at correctly identifying both the pre-epileptic state (“PRE”) and other brain activities (“OTHER”), with a very low number of misclassifications for each category.

The confusion matrix in Figure 6 is related to Experiment 16, which used the EfficientNetV2 model on the D2 dataset (images created with the Mexican Hat wavelet transform). It exhibits following values:

- True Positives (PRE correctly predicted as PRE): 120
- True Negatives (OTHER correctly predicted as OTHER): 189
- False Positives (OTHER incorrectly predicted as PRE): 11
- False Negatives (PRE incorrectly predicted as OTHER): 80

The confusion matrix in Figure 6 indicates a mixed performance. While the model is correctly identifying the “OTHER” class (with only 11 false positives), there are several misclassification with the “PRE” class. As a matter of fact, it incorrectly classifies 80 pre-epileptic instances as “OTHER,” resulting in a high number of false negatives. This is a critical issue for an early-detection system, as it means many pre-seizure states would be missed.

**Figure 7:**
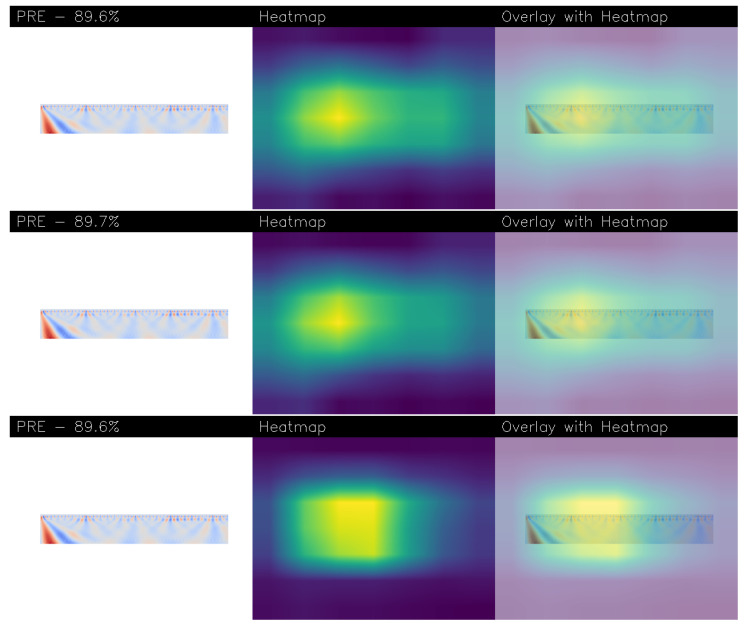
An example of explainability related to the same patient correctly labelled with the PRE label obtained from the model built from Experiment 5. At the top there is the explainability obtained with Gradcam++, in the middle is that obtained with Score-CAM, while at the bottom is that obtained with Score-CAM Fast.

**Figure 8:**
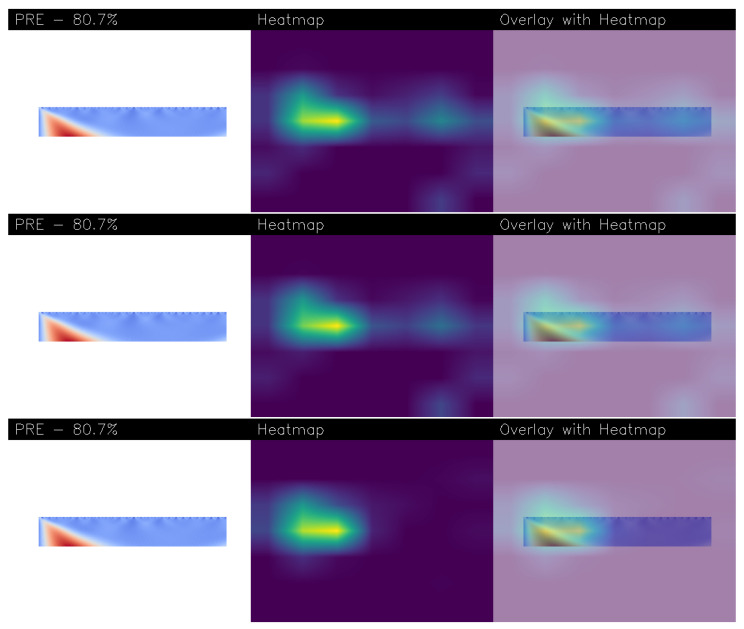
An example of explainability related to the same patient correctly labelled with the PRE label obtained from the model built from Experiment 16. At the top there is the explainability obtained with Gradcam++, in the middle is that obtained with Score-CAM, while at the bottom is that obtained with Score-CAM Fast.

Figure 7 and Figure 8 shows several examples of explainability aimed to understand the decision-making process of models obtained from Experiments 5 and 16, visually explaining which features within the input images led to a “PRE” (pre-epileptic seizure) classification. We also found that in the worst-performing models, the explainability algorithms did not highlight areas of interest i.e., the heatmaps were completely purple, meaning no area of the images was particularly interesting for prediction.

Each figure presents three rows, with each row dedicated to a different explainability technique: Grad-CAM++, Score-CAM, and Score-CAM Fast.

Within each row, a triplet of images is shown: each row is related to a different explainability algortithm, the first one is related to Gradcam++, the second one to the Score-CAM and the last one to the Score-CAM Fast. Thus, in each row, the first image is the original wavelet-transformed EEG plot fed to the network. The central image is a heatmap generated using the VIRIDIS colormap, which translates the model’s focus into a spectrum of colors. Bright yellow areas signify the regions the network deemed most critical (and thus most of interest) for its decision, while green and teal indicate moderate importance, and dark blue or purple tones represent the least influential parts. The final image in the triplet overlays this heatmap onto the original plot, precisely identifying the area of the input that the network found to be symptomatic of the pre-epileptic condition. Additionally, each analysis includes the model’s confidence percentage for the “PRE” prediction.

Figure 7 specifically details the explainability for the DenseNet model from Experiment 5. The input image, derived from a Morlet wavelet transform, shows a sharp, high-frequency energy burst colored in red at the very beginning of the time-frequency plot. All three explainability methods generate heatmaps with a concentrated yellow hotspot that aligns perfectly with this initial transient event. This remarkable consistency across Grad-CAM++, Score-CAM, and Score-CAM Fast demonstrates that the model has reliably learned to associate this specific early-onset, high-energy feature with the pre-epileptic state. The fact that all methods localize the same area strongly suggests the model’s decision is robust and based on a distinct, identifiable pattern.

Figure 8 provides an example of explainability results for the EfficientNetV2 model from Experiment 16. This image, generated using the Mexican Hat wavelet, displays a broader, more gradual increase in energy at the start of the signal, visible as a triangular red region on the left. Once again, the heatmaps from all three CAM techniques are in agreement, placing their bright yellow core directly over this initial energy surge. This indicates that the EfficientNetV2 model, when analyzing the Mexican Hat representation, has identified this ramp-up in signal energy as the key symptomatic feature. The consensus among the different explainability methods reinforces the trustworthiness of the model’s focus, showing that it consistently pinpoints the same discriminative region to make its prediction.

Thus, in the explainability analysis, three CAM algorithms—Grad-CAM++, Score-CAM, and Score-CAM Fast are employed to interpret and visualize the decision-making behavior of the proposed deep learning models. The generated heatmaps from all three techniques consistently emphasized the same salient regions within the analyzed EEG-derived images, thereby validating the model’s ability to focus on clinically relevant areas associated with epileptic seizure activity. This consistent localization across different CAM approaches confirms the robustness and reliability of the model’s internal representations. Moreover, by visually correlating the model’s attention with medically meaningful regions, the proposed method enhances explainability and supports clinical decision-making. Such transparency not only allows clinicians to verify the rationale behind automated predictions but also fosters greater confidence and trust in the deployment of AI-assisted diagnostic systems in real-world medical environments.

## 4. Discussion

The experimental results obtained in this study highlight the effectiveness of the proposed explainable deep learning framework for epileptic seizure detection based on EEG-derived audio signals and wavelet transformations. Among the tested CNN architectures, DenseNet and EfficientNetV2 achieved the best overall performance, with accuracy values of 0.922 and 0.867 for the D1 (Morlet wavelet) and D2 (Mexican Hat wavelet) datasets, respectively. These results confirm that feature-rich architectures with dense connectivity or compound scaling are particularly suitable for modeling complex time–frequency patterns extracted from EEG signals.

The observed differences between performances on the Morlet and Mexican Hat wavelet datasets indicate that the choice of wavelet function directly influences the discriminative power of the extracted representations. The Morlet wavelet, characterized by strong time–frequency localization, yielded superior results, likely because it captures transient high-frequency oscillations typical of pre-ictal activity. Conversely, the Mexican Hat wavelet provided smoother and more global representations, emphasizing amplitude modulations rather than transient oscillations. Together, these complementary characteristics demonstrate that different wavelets can reveal distinct aspects of neural dynamics, and combining them may further enhance seizure prediction performance in future studies.

We are aware that the absence of statistical analysis means that the results cannot be formally tested for significance or generalizability. Consequently, conclusions drawn from the study should be considered preliminary.

From the explainability perspective, the use of Grad-CAM++, Score-CAM, and Score-CAM-Fast consistently confirmed the reliability of the trained models. All three techniques localized similar regions of interest within the time–frequency images, focusing on areas characterized by strong spectral activity that corresponded to pre-epileptic patterns. The coherence among different CAM methods reinforces the interpretability of the models, indicating that the learned features are not spurious but clinically relevant. This aspect is crucial for the potential integration of deep learning models into clinical decision-support systems, where transparency and interpretability are essential for user trust.

Despite the encouraging results, the proposed method presents some limitations. First, the transformation of EEG signals into audio and subsequently into wavelet-based images, while interesting, may lead to partial loss of fine-grained temporal information inherent in the raw EEG signals. Second, the study relies on a single publicly available dataset, which limits the generalizability of the results across different patient populations, recording conditions, and hardware setups. Additionally, although the use of Grad-CAM++, Score-CAM, and Score-CAM-Fast provides valuable interpretability, these methods are qualitative and may not fully capture the causal relevance of highlighted regions.

Overall, the results suggest that transforming EEG signals into audio and subsequently into wavelet-based time–frequency representations provides a promising approach for explainable seizure detection. The combination of signal transformation and explainability not only improves model transparency but also aligns with the clinical need for trustworthy deep learning-based methods.

As a matter of factm the peak of DenseNet performance (accuracy 0.922, AUC 0.976) and EfficientNetV2’s strong results (accuracy 0.867, AUC 0.946) indicate that the proposed wavelet–CNN pipeline yields classification quality that clearly surpasses many traditional machine-learning approaches historically used for seizure detection, which typically rely on handcrafted features and report lower generalization across patients [11]. Compared with more recent deep-learning efforts, the proposed method performs competitively: the high AUC values demonstrate very good discriminative capability on the chosen intracranial EEG dataset, and the consistently better performance on Morlet-derived images suggests that time–frequency representations with strong time–localization are particularly beneficial for pre-ictal pattern recognition. Direct numeric comparisons with the recent works cited in this manuscript (e.g., the contemporary deep models and multimodal approaches referenced in [12,18]) are complicated by differences in datasets, class definitions (pre-ictal vs. ictal/inter-ictal splits), preprocessing pipelines, and evaluation protocols, but qualitatively our results place the proposed framework at least on par with, and in some respects superior to, many state-of-the-art methods because it combines both high classification performance and consistent, concordant explainability outputs. Importantly, the explainability analysis (Grad-CAM++, Score-CAM and Score-CAM Fast) provides an additional advantage over many prior studies that report only metrics: the strong agreement among CAM methods lends confidence that the model is focusing on clinically meaningful time–frequency events rather than spurious artifacts, which improves the practical value of the system for clinical decision support. Finally, while the experimental outcomes are encouraging, the single-dataset evaluation limits claims of broad generalizability; future work that applies the same wavelet + CNN + CAM pipeline across multiple publicly available EEG seizure datasets and reproduces these results under standardized protocols will be necessary to confirm that the observed gains hold across recording modalities and patient populations.

## 5. Conclusions and Future Work

In this paper, we proposed an explainable deep learning method for epileptic seizure detection, by exploiting EEG signals processed through audio conversion and wavelet-based transformation. Utilizing the Morlet and Mexican Hat continuous wavelet transforms, two distinct image datasets were generated to capture the time–frequency characteristics of EEG activity. Experimental results indicate that the proposed method achieves interesting performance in the detection of epileptic seizures from EEG data. Among the evaluated models, the DenseNet model obtained the highest accuracy (0.922) on the Morlet-transformed dataset (i.e., the D1 dataset), whereas EfficientNetV2 achieved a lower accuracy (0.867) on the Mexican Hat-transformed dataset (i.e., the D2 dataset). In the explainability analysis, we considered three different CAM algorithms, i.e., Grad-CAM++, Score-CAM, and Score-CAM Fast, by highlighting that all the CAM algorithms highlighted the same areas of the analyzed image, thus confirming the image area of interest for the model and localizing the part of the image relating to the epileptic seizure according to the trained model. In future work, we plan to consider alternative image representations of audio recordings, such as spectrograms, with the aim of further enhancing classification accuracy. Moreover, additional CNN architecture will be considered for model training for epileptic seizure detection. Moreover, future work will explore multimodal fusion of Morlet and Mexican Hat representations, additional CNN architectures, ensemble strategies, and the inclusion of multiple datasets to enhance both the generalizability and reliability of the proposed method. 

## Figures and Tables

**Table 1:** Comparison of Grad-CAM++, Score-CAM, and Score-CAM Fast in terms of gradient order and passes required.

Method	Gradient Order	Passes Required
Grad-CAM++	First-, second-, and third-order	1 backward (higher-order) + 1 forward
Score-CAM	None (gradient-free)	*K* forward passes (per feature map)
Score-CAM Fast	None (gradient-free)	≪K forward passes (subset/approx.)

**Table 2:** Hyper -parameters setting.

Exp.	Model	Image Size	Learning Rate	Epochs	Batch Size	Dataset
#1	AlexNet	224 × 224 × 3	1 × 10^−1^	10	16	D1
#2	LeNet	224 × 224 × 3	1 × 10^−1^	10	16	D1
#3	STANDARD_CNN	224 × 224 × 3	1 × 10^−1^	10	16	D1
#4	MobileNet	224 × 224 × 3	1 × 10^−1^	10	16	D1
#5	DenseNet	224 × 224 × 3	1 × 10^−1^	10	16	D1
#6	EfficientNetV2	224 × 224 × 3	1 × 10^−1^	10	16	D1
#7	ResNet50	224 × 224 × 3	1 × 10^−1^	10	16	D1
#8	VGG16	224 × 224 × 3	1 × 10^−1^	10	16	D1
#9	VGG19	224 × 224 × 3	1 × 10^−1^	10	16	D1
#10	GoogleNet	224 × 224 × 3	1 × 10^−1^	10	16	D1
#11	AlexNet	224 × 224 × 3	1 × 10^−1^	10	16	D2
#12	LeNet	224 × 224 × 3	1 × 10^−1^	10	16	D2
#13	STANDARD_CNN	224 × 224 × 3	1 × 10^−1^	10	16	D2
#14	MobileNet	224 × 224 × 3	1 × 10^−1^	10	16	D2
#15	DenseNet	224 × 224 × 3	1 × 10^−1^	10	16	D2
#16	EfficientNetV2	224 × 224 × 3	1 × 10^−1^	10	16	D2
#17	ResNet50	224 × 224 × 3	1 × 10^−1^	10	16	D2
#18	VGG16	224 × 224 × 3	1 × 10^−1^	10	16	D2
#19	VGG19	224 × 224 × 3	1 × 10^−1^	10	16	D2
#20	GoogleNet	224 × 224 × 3	1 × 10^−1^	10	16	D2

**Table 3:** Experimental results obtained with the D1 and the D2 datasets.

Exp.	Model	Loss	Accuracy	Precision	Recall	F-Measure	AUC	Execution Time
#1	AlexNet	1.220	0.564	0.564	0.564	0.564	0.748	0:10:04
#2	LeNet	0.693	0.5	0.5	0.5	0.5	0.5	0:08:54
#3	STANDARD_CNN	0.689	0.5	0.5	0.5	0.5	0.659	0:09:35
#4	MobileNet	0.373	0.865	0.865	0.865	0.865	0.936	0:12:00
#5	DenseNet	0.229	0.922	0.922	0.922	0.922	0.976	0:22:36
#6	EfficientNet	0.323	0.867	0.867	0.867	0.867	0.946	0:21:00
#7	ResNet50	0.543	0.845	0.845	0.845	0.845	0.911	0:16:46
#8	VGG16	0.693	0.5	0.5	0.5	0.5	0.5	0:15:52
#9	VGG19	0.693	0.5	0.5	0.5	0.5	0.5	0:16:37
#10	GoogleNet	0.693	0.5	0.5	0.5	0.5	0.5	0:10:50
#11	AlexNet	0.411	0.834	0.834	0.834	0.834	0.894	0:09:14
#12	LeNet	0.693	0.5	0.5	0.5	0.5	0.5	0:09:08
#13	STANDARD_CNN	0.687	0.697	0.697	0.697	0.697	0.736	0:09:50
#14	MobileNet	0.613	0.779	0.779	0.779	0.779	0.869	0:12:37
#15	DenseNet	0.493	0.82	0.827	0.827	0.827	0.890	0:22:14
#16	EfficientNet	0.323	0.867	0.867	0.867	0.867	0.946	0:21:00
#17	ResNet50	0.768	0.790	0.790	0.790	0.790	0.854	0:16:51
#18	VGG16	0.693	0.5	0.5	0.5	0.5	0.5	0:13:01
#19	VGG19	0.693	0.5	0.5	0.5	0.5	0.5	0:16:34
#20	GoogleNet	0.692	0.5	0.5	0.5	0.5	0.538	0:10:37

## Data Availability

We utilized a publicly available dataset (https://doi.org/10.5281/zenodo.10808054, (accessed on 23 December 2025).
